# Possible mechanisms of host resistance to *Haemonchus contortus* infection in sheep breeds native to the Canary Islands

**DOI:** 10.1038/srep26200

**Published:** 2016-05-20

**Authors:** Zhengyu Guo, Jorge Francisco González, Julia N. Hernandez, Tom N. McNeilly, Yolanda Corripio-Miyar, David Frew, Tyler Morrison, Peng Yu, Robert W. Li

**Affiliations:** 1 Department of Electrical and Computer Engineering & TEES-AgriLife Center for Bioinformatics and Genomic Systems Engineering (CBGSE) Texas A&M University, College Station, TX 77843, USA; 2 Department of Animal Pathology, Faculty of Veterinary Medicine, University of Las Palmas de Gran Canaria, Las Palmas, Spain; 3 Moredun Research Institute, Midlothian EH26 0PZ, Scotland, UK; 4 United States Department of Agriculture, Agriculture Research Service (USDA-ARS), Animal Genomics and Improvement Laboratory, Beltsville, MD 20705, USA

## Abstract

*Haemonchus contortus* appears to be the most economically important helminth parasite for small ruminant production in many regions of the world. The two sheep breeds native to the Canary Islands display distinctly different resistant phenotypes under both natural and experimental infections. Canaria Hair Breed (CHB) tends to have significantly lower worm burden and delayed and reduced egg production than the susceptible Canaria Sheep (CS). To understand molecular mechanisms underlying host resistance, we compared the abomasal mucosal transcriptome of the two breeds in response to *Haemonchus* infection using RNAseq technology. The transcript abundance of 711 and 50 genes were significantly impacted by infection in CHB and CS, respectively (false discovery rate <0.05) while 27 of these genes were significantly affected in both breeds. Likewise, 477 and 16 Gene Ontology (GO) terms were significantly enriched in CHB and CS, respectively (*P* < 1.0 × 10^−4^). A broad range of mechanisms have evolved in resistant CHB to provide protection against the parasite. Our findings suggest that readily inducible acute inflammatory responses, complement activation, accelerated cell proliferation and subsequent tissue repair, and immunity directed against parasite fecundity all contributed to the development of host resistance to parasitic infection in the resistant breed.

The intestinal worm *Haemonchus contortus* is arguably the most economically important helminth parasite for small ruminant production in many regions of the world. As a voracious blood feeder residing in the mucosal layer of the abomasum, *H. contortus* causes anaemia and hyper-gastrinaemia and alters abomasal secretion. *H. contortus* infection results in reduced growth, compromised reproduction, and elevated mortality, due to its ubiquitous distribution and severe pathogenicity. Consequently, *H. contortus* parasitism represents the primary constraint to profitable production of sheep and goats worldwide.

Over the past years, the rapid emergence of drug-resistant *H. contortus* strains and increasing demands by consumers for inexpensive organic meat and milk products with less drug residues have spurred research on the development of anthelmintic-independent parasite control strategies, such as vaccines^1^ and novel biologics, nutrient supplements and bioactive compounds, and selective breeding. Among them, selectively breeding sheep and goats with abilities to better resist parasitic infections appears to be a solution to sustainable small ruminant production.

Differences in resistance and susceptibility to parasitic infections between sheep breeds have been long documented^2^. Over the decades, comparative studies have identified at least 19 sheep breeds displaying varying degrees of resistance to parasitic infections^3^. For example, St. Croix lambs shed significantly fewer eggs and harbor 99% fewer worms in the abomasum than the age-matched Dorset lambs during both natural and experimental infections^4^. Locally-adapted breeds such as Santa Ines sheep of Brazil have significantly reduced worm burdens and fewer nodular lesions under natural infections than Suffolk and Ile de France lambs on the same pasture^5^. In Europe, resistance against *H. contortus* is better developed in Merinoland sheep than in Rhon sheep^6^. Red Maasai sheep have been shown to be more resistant to *Haemonchus* infection than the South African Dorper breed during natural exposure to parasites in Kenya^7^. Moreover, resistance to parasite infection has a significant genetic component. The contribution of the host genome and genetics has been estimated. For example, additive genetic variation accounts for approximately 30% of the overall variation for parasitic infection^8^. The resistance traits are often polygenic in nature and not influenced by genes with major effects^9^. Nevertheless, estimates of heritability for parasite indicator traits in small ruminants are phenotype-dependent, ranging from 0.11 to 0.40 for transformed fecal egg counts (EPG) and 0.19 to 0.26 for packed cell volume (PCV) in German Rhon sheep^10^. In addition, the host age plays a role. A good example is that in Scottish Blackface lambs at the end of the first grazing season, the heritability of adult worm length is very strong at 0.62^8^. While many efforts have been made to identify genetic variants associated with parasite resistance and tolerance in sheep breeds^11^,^12^,^13^, molecular mechanisms and biological pathways underlying host resistance to parasitic infections in sheep remain largely unknown.

Due to unique geographical characteristics of the Canary Islands, indigenous sheep breeds have been exploited by local farmers for centuries. Among them, the Canaria Hair Breed (CHB) and Canaria sheep (CS) are predominately raised for the production of meat and milk, respectively. Previous studies demonstrate that CHB constantly displays better resistance phenotypes to *H. contortus* infection than CS, including significantly lower levels of fecal egg counts, fewer adult worm counts, lower number of eggs in utero and female worm stunting^14^. Further studies^15^ identified significant negative correlations between two effector cells, eosinophils and γδ/WC1+ T cells, and parasite fecundity in CHB, suggesting that inter-breed difference in regulating immune responses affects *Haemonchus* infection. In this study, we conducted a RNA-seq based comparative transcriptome analysis in the two indigenous breeds and attempted to understand the molecular basis underlying host resistance.

## Results

### Parasitology

**Figure 1:**
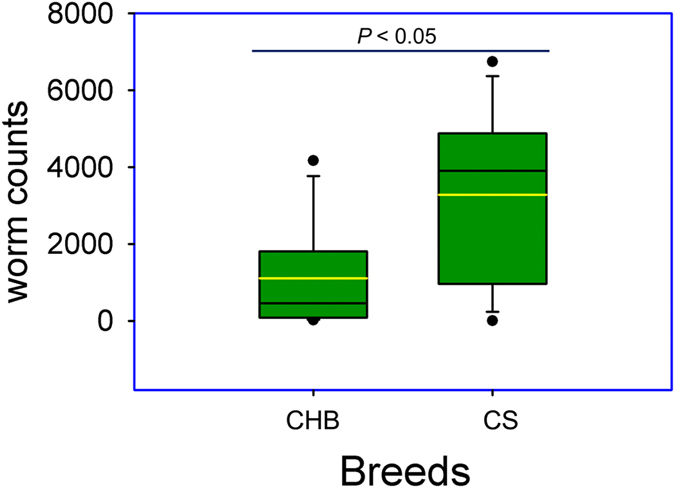
Differences in worm counts between resistant and susceptible sheep breeds under experimental *Haemonchus contortus* infection. Boxes denote the inter-quartile range between the 1^st^ and 3^rd^ quartiles (25 and 75%, respectively). Black line: mean; Yellow line: median. CHB: Canaria Hair Breed (resistant). CS: Canaria sheep (susceptible).

The total worms recovered from the infected groups of CHB and CS were 1,109.75 (±1,547.73, SD) and 3,280.50 (±2,398.03), respectively. The difference is statistically significant (*P* < 0.05, Fig. 1). Neither *Haemonchus* worms nor fecal eggs were recovered from the uninfected group of either breed, as expected. EPG values detected from infected CS sheep were 262.50 ± 287.54 (mean ± SD) while no fecal eggs were detectable in the infected group of CHB sheep at 20 days post infection (dpi). No parasite eggs in either group prior to the experimental challenge were observed.

### *Haemonchus* infection induced distinctly different transcriptome patterns in the abomasal mucosa of CHB and CS breeds

**Figure 2:**
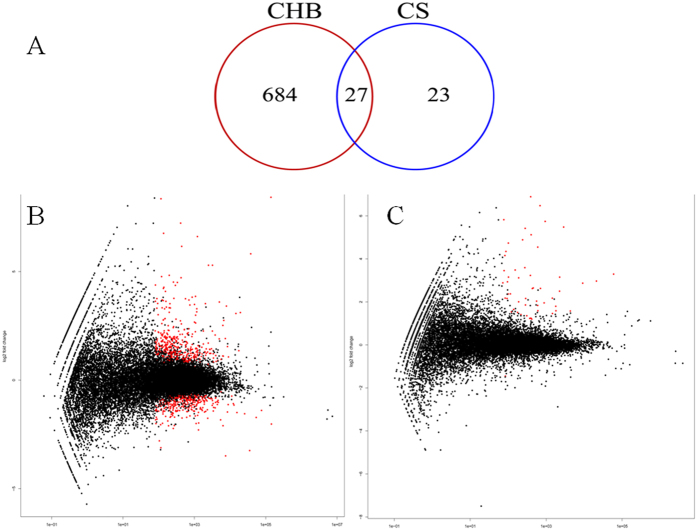
(**A**) Venn Diagram showing the number of genes with significant differences in transcript abundance induced by infection in two sheep breeds compared to their respective uninfected controls at a false discovery rate (FDR) cutoff <0.05. CHB: Canaria Hair Breed (resistant). CS: Canaria Sheep (susceptible). (**B**,**C**) Scatter plot of log_2_ ratio (fold change) vs mean. The red color indicates genes detected as differentially expressed between the infected group and uninfected controls at a false discovery rate (FDR) < 0.05 in resistant Canaria Hair Breed (**B**) and susceptible Canaria Sheep (**C**).

**Figure 3:**
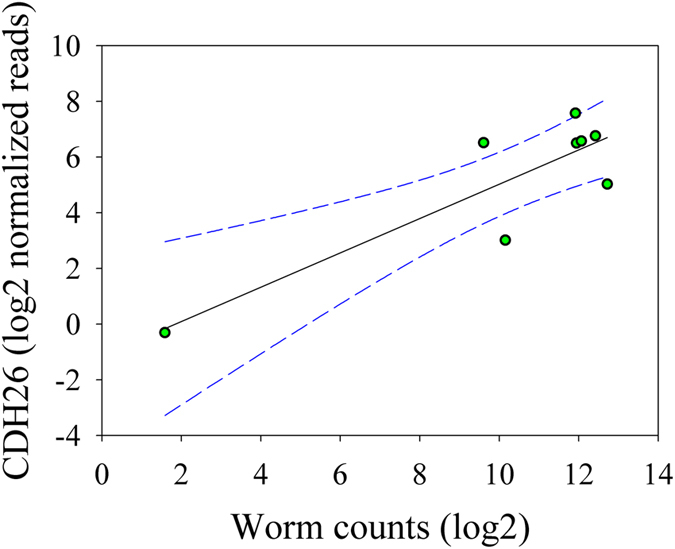
Nonlinear regression between worm counts and normalized transcript abundance per million mapped reads of the gene cadherin 26 (CDH26) in susceptible Canaria Sheep (CS). Dotted lines: 95% confidence interval.

In this study, approximately 79.91% of raw reads (±7.08%; SD) were uniquely mapped to the ovine genome. Compared to their respective uninfected controls, the numbers of genes significantly impacted by infection in CHB and CS breeds at a stringent cutoff value (false discovery rate or FDR < 0.05), were 711 and 49, respectively (Fig. 2). The abundance of 27 genes was significantly changed by infection in both breeds (Table 1). Among them, 25 genes, such as arachidonate 15-lipoxygenase (ALOX15), collagen, type VI, α5 (COL6A5), and serglycin (SRGN), were significantly upregulated while the expression of transthyretin (TTC) was repressed by infection. Intriguingly, the transcript abundance of cadherin 26 (CDH26) was significantly induced by infection in both breeds (adjusted *P* value or FDR < 1.63 × 10^−10^); and is strongly correlated with worm counts only in CS (Fig. 3). However, infection had a bidirectional impact on the transcript abundance of a uncharacterized gene containing a unknown microRNA (ENSOARG00000023771), which was significantly upregulated in CHB but downregulated in CS. The genes significantly impacted by infection only in CS included mast cell proteinase-3, γ-glutamyltransferase 5 (GGT5), CD163 as well as those involved in smooth muscle contraction, such as tropomyosin (TPM2), myosin, light chain 9, regulatory (MYL9), and calponin 1, basic, smooth muscle (CNN1).

Among the genes significantly impacted by infection in CHB sheep, several cytokine receptors and chemokines were strongly upregulated. Notable, the transcript of IL17 receptor beta (IL17RB) was 14.4 fold higher in infected animals than uninfected controls in CHB. IL2 receptor beta (IL2B) was also upregulated. Similarly, chemokine CXC ligand 12 (CXCL12) and chemokine (CXC motif) receptor 6 (CXCR6) were upregulated by infection in CHB. Among the well-known Th2 cytokines, the expression of IL6, IL10 and IL13 was upregulated by infection in both breeds. Moreover, while the extent of upregulation of IL6 by infection remained similar in both breeds (~6.8 fold), overexpression of both IL10 and IL13 mRNA molecules was more profound in the resistant breed (CHB) than in CS. On the other hand, the IL5 mRNA was upregulated by infection in CS but barely detectable in CHB at the sequencing depth in this study. The IL4 expression followed the similar trend: it was upregulated approximately 9 fold by infection in CS but was barely detectable in CHB. However, the IL9 mRNA level remained unchanged by infection in both breeds.

Several genes involved in arachidonic acids metabolism, including eicosanoids metabolism, were significantly impacted by infection, such as arachidonate 5-lipoxygenase (ALOX5) and its activating protein (ALOX5P), prostaglandin reductase 1 (PTGR1), prostaglandin-endoperoxide synthase 1 (prostaglandin G/H synthase and cyclooxygenase) (PTGS1, COX1), and thromboxane A synthase 1 (TBXAS1), were all strongly upregulated by infection in CHB. In addition, at least 11 genes implicated in complement activation were significantly impacted by infection in CHB, such as complement factor properdin (CFP, 2.8 fold), complement component 7 (C7, 4.2 fold), and complement factor I (CFI, 12.1 fold). Other known genes involved in protective immunity to helminth infection strongly upregulated by infection in CHB included amphiregulin (AREG, 2.2 fold), granzyme genes A and B (GZMA and GZMB, 6.8 and 12.9 fold, respectively).

41 of the 711 genes significantly impacted by infection in CHB are related to extracellular matrix (ECM, Table 2). Of them, fibronectin 1 (FN1) was strongly upregulated. At least ten collagen genes were significantly upregulated, such as those from Type I, Type III, Type V, Type VI, and Type XII (Table 2). For example, the expression of collagen, type VI, alpha 5 (COL6A5) and collagen, type XII, alpha 1 (COL12A1) was increased 20.8 and 2.4 fold, respectively in CHB, compared to the uninfected controls. Likewise, matrix metallopeptidase 1 (MMP1), MMP2, and MMP14 were significantly up-regulated while the transcript of MMP11 was repressed by infection. Furthermore, several cell adhesion molecules, including integrins, lectins, and cadhesion, were strongly upregulated by infection in CHB, such as conglutinin-like (COLEC8, 451.7 fold), integrin, α11 (ITGA11, 3.4 fold), and lectin, galactoside-binding, soluble, 15 (LGALS15, 340.1 fold).

Of note, approximately 15% of the genes significantly impacted by infection are cell-cycle related. The expression of these cell cycle related genes was predominantly enhanced by *Haemonchus* infection in CHB. As Table 3 shows, at least 92 genes were significantly upregulated by infection, such as cyclin A2 (CCNA2), cyclin B3 (CCNB3), various centromere proteins (CENPL, CENPN, CENPT, and CENPW) and kinesin family (KIF) members, and at least 5 minichromosome maintenance complex (MCM) components (MCM3, MCM4, MCM5, MCM6, and MCM10). Nevertheless, the infection was also able to repress cell cycle related genes, such as cyclin G1 (CCNG1), regulator of cell cycle (RGCC), and synaptonemal complex protein 3 (SYCP3). Moreover, at least five transcription factors, such as the oncogene MYB, SMAD family members 6 and 9 (SMAD6 and SMAD9), and histone decetylase 5 (HDAC5), were significantly affected by infection in CHB.

Intriguingly, four genes known to regulate abomasal acid secretion and gastric function^16^ were downregulated by *Haemonchus* infection in CHB, including ATPase, H+/K+ exchanging, alpha polypeptide (ATP4A), progastricsin (pepsinogen C, PGC), appetite-regulating hormone precursor (GHRL), and forkhead box A2 (FOXA1). However, the transcript abundance of these four genes remained unchanged by infection in CS.

**Figure 4:**
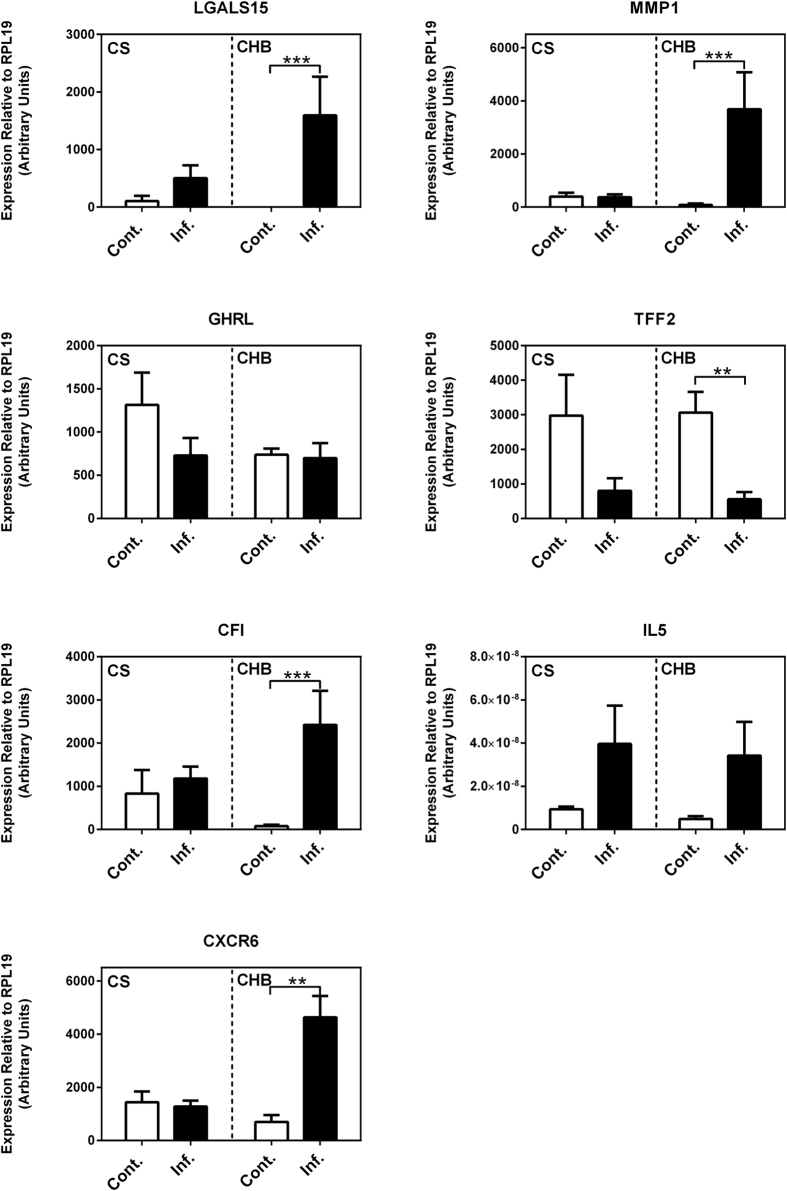
Real-Time RT-PCR analysis (qPCR) of selected genes. Relative expression levels calculated from standard curves were normalized to the endogenous control gene RPL19. Numbers represent mean values plus standard error. Cont.: uninfected controls; Inf.: 20 days post infection by *Haemonchus contortus.* CS: Canaria Sheep; CHB: Canaria Hair Breed. CFI: Complement factor I; CXCR6: Chemokine (C-X-C motif) receptor 6; GHRL: Ghrelin/obestatin prepropeptide; LGALS15: Galectin 15; IL5: Interleukin 5. MMP1: Matrix metallopeptidase 1; TFF2: Trefoil factor 2. **P < 0.001; ***P < 0.0001.

**Figure 5:**
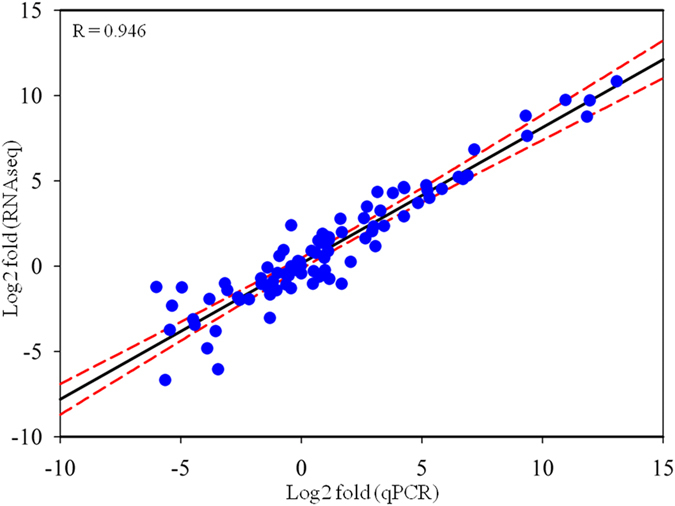
Linear regression analysis of fold changes calculated from qPCR and RNAseq analysis. Blue dots represent log_2_ transformed fold change values of a single gene in an infected sample obtained from qPCR (X-axis) and RNAseq analysis (Y-axis). Dashed lines: 99% Confidence Interval. R: correlation coefficient.

The RNAseq results of selected genes were validated by real-time RT-PCR (Fig. 4). For example, the expression of CFI, CXCR6, LGALS15, and MMP1 was significantly upregulated while TFF2 mRNA level was significantly repressed by infection only in the resistant breed (CHB), in a good agreement with the RNAseq analysis. A strong correlation in log_2_ transformed fold values between the two platforms, qPCR and RNAseq, was evident (a correlation coefficient R = 0.946; Fig. 5).

### Gene Ontology (GO) implicated in host resistance

**Figure 6:**
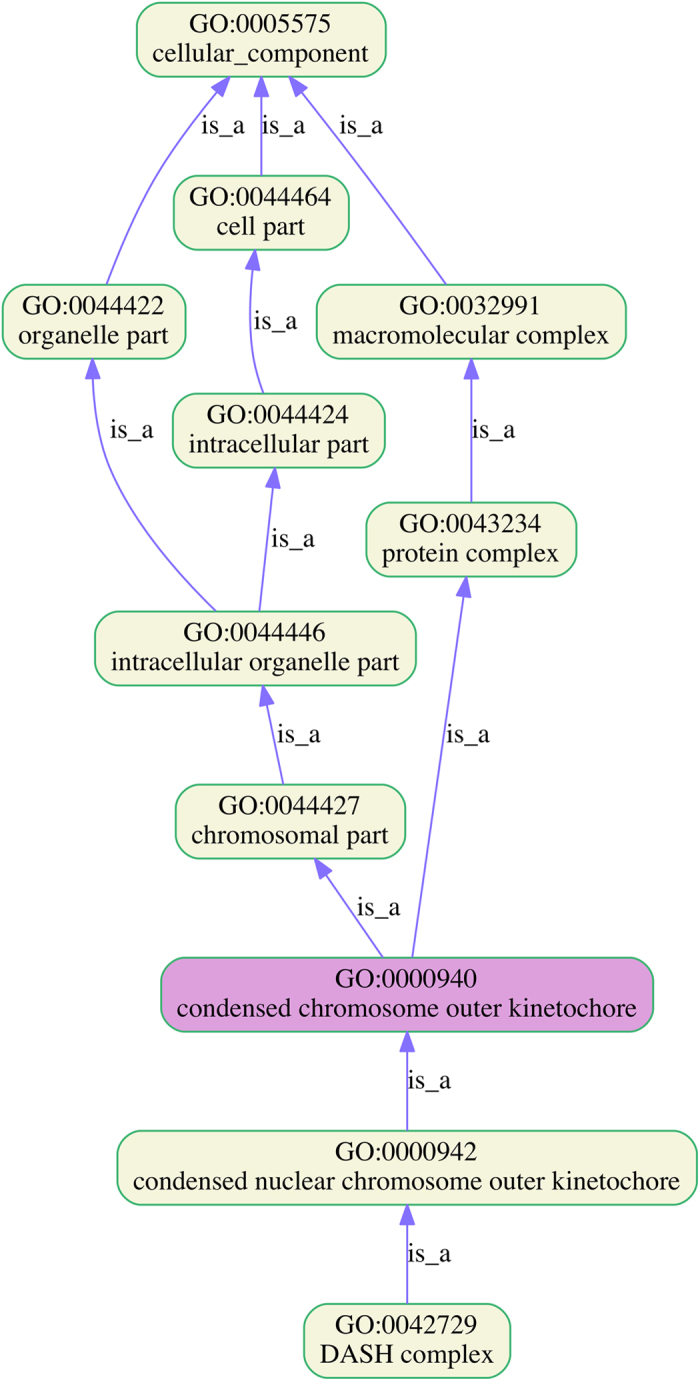
Gene Ontology (GO) lineage relations. The Cellular Component Ontology term GO:0000940 (condensed chromosome outer kinetochore) significantly enriched in resistant Canaria Hair Breed (CHB, adjusted *P* value < 2.30 × 10^−4^).

Among 477 and 16 GO terms significantly enriched in CHB and CS at a *P* value cutoff 1.0 × 10^−4^, respectively, five were significantly enriched in both breeds (Table 4). Select GO terms that may be implicated in the development of host resistance to *Haemonchus* infection are listed in Table 5. Several GO related to complement activation (both classical and alternative pathways) and its regulation were significantly enriched only in CHB. Numerous cell cycle related GO were significantly enriched as well (Fig. 6). GO related to secretory granule and gastric acid secretion were also enriched, suggesting that the ability to regulate secretory and gastric function of the host may be involved in the development of host resistance. Furthermore, the regulation of inflammation at the site of infection (mucosa), including arachidonic acid metabolism, cyclooxygenase pathway, and positive regulation of MAPK cascade, as well as leukocyte migration were also implicated in host resistance. On the other hand, four of the 11 GO unique to CS were related to muscle contraction.

## Discussion

Parasite resistance refers to the ability of the host to avert infection, resulting in reduced worm burden^17^. Numerous factors affect this trait. Among them, host genetics play a predominant role in controlling the development of resistance while host sex, age, and prior exposure are also important^18^. Differences in parasite resistance and susceptibility existing in various sheep breeds have been long recognized^3^. Moreover, inter- and intra-host variations in resistance are evident in certain sheep populations^18^. Identifying genetics components controlling inter-, and intra-breed differences in parasite resistance has both pragmatic and theoretical implications. Towards this end, numerous efforts have been made over the decades to unravel genes and/or genetic variants responsible for resistance, partially driven by strong desires to breed farm animals with strong resistance traits. Traditional QTL analysis and Genome-wide Association Studies (GWAS) have led to reports of dozens of QTL or markers on almost every ovine chromosome that are associated with various resistance phenotypes, such as fecal egg counts, packed cell volume, and parasite-specific antibody titers^9^,^11^,^13^. Nevertheless, the development of parasite resistance relies upon the precise control of expression of the host genome. Understanding these regulatory elements will be crucial towards unraveling their functional relevance. As a result, while much progress has been made to identify genes associated with nematode resistance in sheep during the past few years^19^,^20^, an in-depth comparison and characterization of transcriptome responses of various breeds and populations, especially those local indigenous breeds harboring varying degrees of parasite resistance and susceptibility, is urgently needed.

The two indigenous breeds of sheep native to the Canary Islands, CHB and CS, display unique and distinct differences in parasite resistance and susceptibility. When co-grazing together on the same pasture under natural infections, differences in fecal trichostrongylid egg counts between CHB and CS are consistently observed^14^. Under experimental infections with *H. contortus*, CHB has a significantly lower, by approximately 50%, worm burden than CS, a undeniable trait of parasite resistance^14^,^15^, which is confirmed in this study. Moreover, worms recovered in CHB tend to have significantly shorter body length than those in CS. A significantly lower EPG value is consistently observed in the feces of CHB sheep than those of CS animals during experimental infection. For example, at 27dpi, the mean EPG in CS is 5 fold higher than in CHB^14^. CHB sheep not only shed significantly fewer parasite eggs but also tend to have a delayed egg production, indicating an anti-fecundity effect of the immune response in this breed. The results from this study show that at 20 dpi, no parasite eggs were recovered in the feces of infected CHB animals while EPG in the feces of infected CS sheep reached 262.50 (±287.54, SD). This observation is in agreement with the previous findings^14^. *Haemonchus contortus* infection generally elicits a potent Th2 immune response in small ruminants. A strong upregulation of several well-known Th2 cytokines by infection in CHB were observed in this study. Previous studies in the Canary Island breeds suggest that divergence in immune response mechanisms exist between CHB and CS. Among various immune cells, abomasal eosinophil numbers are 2 fold higher in CHB than in CS, suggesting that CHB sheep may have developed abilities for enhanced recruitment of eosinophils to the site of infection (abomasal mucosa). Furthermore, CHB sheep have evolved mechanisms attacking the adult stage of the *Haemonchus* parasite, especially its reproduction, as evidenced by the fact that fecundity is negatively correlated with eosinophils and γδ T cells in the abomasal mucosa^15^. However, the precise molecular mechanisms of the parasite resistance in CHB breed remain largely unclear.

In this study, we identified a total of 477 and 16 Gene Ontology (GO) terms that are significantly enriched in the transcriptome of resistant and susceptible sheep breeds in responses to *Haemonchus* infection, respectively. Among them, only five enriched GO were shared by both breeds. These GO, including leukotriene metabolic process, eicosanoid biosynthesis process, adaptive immune response, and unsaturated fatty acid biosynthesis, likely represents the basic mechanisms of host immune responses to helminth infection in sheep. Indeed, local inflammatory responses are known to be involved in the development of host resistance^21^. The enriched GO unique to the susceptible CS breed were predominantly muscle contraction-related. In cattle, our previous results suggest that smooth muscle hypercontractility induced by primary infection of the intestinal worm *Cooperia oncophora* represents an important aspect of host responses^22^, as in several other host-parasite systems^23^. In rodent models, helminth infection results in an increase in thickness of jejunal smooth muscle layers. Other studies also support the idea that enhanced muscle contractility appears to be associated with more rapid worm expulsion and stronger host immune responses^24^. In addition, granzyme-mediated apoptotic signaling pathway (GO:0008626) may play an important role in protecting the host from *H. contortus* infection in the susceptible CS breed.

Complement activation as one of the earliest events in host immune responses to helminth infection plays an important role in the development of host resistance^25^. At least 11 complement related genes, such as CFI and C7, were significantly impacted by infection in the resistant CHB breed compared to uninfected controls while none of these genes were affected by infection in the susceptible breed. As a result, both classical and alternative complement pathways appeared to be activated in the resistant breed. Furthermore, two GO molecular functions related to C5a (GO:0031714) and C5L2 anaphylatoxin chemotactic receptor binding (GO:0031715) were significantly enriched in the resistant breed. It is conceivable that these peptides play a critical role in subsequent recruitment of effector cells, such eosinophils and mast cells, to the site of infection.

Intriguingly, approximately 15% of the 711 genes whose transcript abundance were significantly altered by infection in the resistant breed were cell cycle related. The vast majority of these genes were significantly upregulated (Table 3). These genes included several cyclins, minichromosome maintenance complex components, and various kinesin family members (Table 3). In addition, a large class of genes significantly impacted in the resistant breed was ECM related (Table 2). ECM related genes are required during the classical stages of wound repair, inflammation, new tissue formation, and remodeling^26^. Previous studies identified essential roles of Th2 cytokines in limiting tissue damage during helminth infection in rodent models, especially the involvement of IL17 in the early stage of tissue repair via its role in neutrophil recruitment^27^. In this study, the transcript abundance of IL17RB was increased approximately14 fold by *Haemonchus* infection only in the resistant breed. Of note, upregulation of Th2 cytokines IL10 and IL13 by infection was more profound in CHB than in CS. Together, our findings suggest that the accelerated tissue repair ability, likely mediated by Th2 cytokines, has evolved in the resistant CHB breed.

Recently, a significant SNP marker on ovine chromosome (OAR) 6 was reported to affect one of key resistant phenotypes in sheep, EPG^9^. This maker explains approximately 4% of the variance observed for EPG. It is suggested that there may exist up to 3 QTL within the 5 Mb region of this locus (73.1–78.3 Mb), in addition to a fourth QTL at 55.9–62.6 Mb on OAV6. Several earlier reports also indicate the presence of the QTL lined to EPG in various sheep breeds^28^,^29^. Among 21 differentially expressed genes located on OAV6 identified in our study in CHB breeds, at least 5 genes are located within 15 Mb of this marker. The expression of four genes were significantly induced whereas one, albumin, was repressed by infection. Of note, mast cell stem cell factor (SCF) receptor KIT gene (chr6:70189728:70234612) is the closest to the SNP marker. Two major receptors, KIT and the high affinity receptor for IgE, are responsible for regulating various mast cell functions, including chemotaxis, proliferation, apoptosis, and cytokine releases^30^. The critical roles of mast cells in host immune responses to helminth infection have long been recognized^31^. Neutralization of KIT and its ligand, SCF, using monoclonal antibodies completely abrogates the mast cell hyperplasia generated by *T. spiralis* infection in mice, resulting in drastically delayed worm expulsion and a reduced mucosal eosinophilia^32^. This finding suggests that KIT plays an important in host-parasite interaction. In the past few years, increasing evidence suggests that the epidermal growth factor like molecule, amphiregulin (AREG), plays critical roles in regulating immunity and inflammation as well as in enhancing host resistance to helminth parasites^33^,^34^. In rodent models, *T. suis* infection increases AREG expression, in parallel with the expression of Th2 cytokines IL4 and IL13^33^. Furthermore, worm clearance is significantly delayed at 14 dpi in AREG deficient mice, which correlates with reduced proliferation of colonic epithelial cells. Recent studies show that AREG is critical for efficient regulatory T cell function^35^ and may play an important role in orchestrating immunity, inflammation, and tissue repair^34^. In this study, AREG transcript abundance was significantly enhanced by infection only in the resistant breed, suggesting that this gene may play an important role in the development of host resistance. It would be intriguing to identify SNPs in both coding and promoter regions of the genes located within or closer to the QTL related to parasite resistance on OAV6, including AREG, and correlate the observed genetic variations with various resistant phenotypes. Moreover, dissecting mechanisms of transcriptional regulation of AREG and understanding how it promotes epithelial cell proliferation and regulates host immunity in the gastrointestinal tract warrant further investigation.

In conclusions, the two sheep breeds native to the Canaria Island displayed a distinct difference in several *Haemonchus contortus* resistant phenotypes under both natural and experimental infections. CHB tends to have significantly reduced worm burden, delayed egg production, and decreased fecal egg yield (counts) than the susceptible Canaria Sheep. A broad range of mechanisms have evolved in resistant CHB to provide protection against *H. contortus*. Readily inducible acute inflammation responses, complement activation, accelerated cell proliferation and subsequent tissue repair, and innate and acquired immunity directly against worm fecundity are likely to contribute to the development of host resistance to gastrointestinal nematode infection in the CHB breed.

## Methods

### Animals and parasitology

Male lambs of CHB (11 animals) and CS breeds (12 animals) were obtained from local farms in the Gran Canaria Island (Spain), weaned, and kept in pens at the Faculty of Veterinary Science, University of Las Palmas de Gran Canaria until they were approximately one year old. The animals were fed with a commercial pelleted sheep ration *ad libitum* and had free access to water throughout the experimental period. The animals were drenched upon arrival with levamisole (Cyber, Fort Dodge, Spain) at the recommended dose (1 ml/10 kg bodyweight) and remained free of parasites (as determined by fecal egg counts) until experimental parasite inoculation. Seven CHB and eight CS animals were inoculated intraruminally with 20,000 *H. contortus* infective L3 larvae. Four age-matched animals of each breed remained uninfected and served as controls. The experimental infection was allowed to progress for 20 dpi. The time point chosen for this study was based on the results from a previous report that the difference in resistance phenotypes, especially mean EPG values, is most profound between the two breeds^14^. Furthermore, no fecal parasite eggs become detectable at this time point in CHB. At 20 dpi, both infected and control animals were sacrificed. EPG values were monitored twice during the experiment, prior to the experimental inoculation and immediately prior to necropsy using the MacMaster technique. Adult worms as well as immature larvae from both contents and the tissue of the abomasum were isolated and counted. The fundic abomasum tissue was then sampled and snap frozen in liquid nitrogen prior to storage at −80 °C until total RNA was extracted. The *Haemonchus* strain used in this trial was initially donated by Drs. Knox and Bartley (Moredun Research Institute, Edinburgh, Scotland) and passaged through successive inoculations in sheep at the premises of the Faculty of Veterinary Science, University of Las Palmas de Gran Canaria (Spain). During the experiment, all animal protocols were approved by the Animal Care and Use Committee of University of Las Palmas per the Institutional Animal Care and Use Committee (IACUC) guidelines. All experimental procedures were carried out in accordance with the approved protocols.

### RNA extraction and sequencing using RNA-seq

Total RNA from fundic abomasal samples of both CHS and CS sheep breeds was extracted using Trizol (Invitrogen, Carlsbad, CA, USA) followed by DNase digestion and Qiagen RNeasy column purification (Qiagen, Valencia, CA, USA), as previously described^22^,^36^. The RNA integrity was verified using an Agilent BioAnalyzer 2100 (Agilent, Palo Alto, CA, USA). High-quality RNA (RNA integrity number or RIN > 7.5) was processed using an Illumina TruSeq RNA sample prep kit following the manufacturer’s instructions (Illumina, San Diego, CA, USA). Pooled RNAseq libraries were sequenced at 2 × 101 bp/sequence read using an Illumina HiSeq 2000 sequencer, as described previously^37^. Approximately 56 million paired-end sequence reads per sample (mean ± SD = 55,945,621 ± 41,305,493.24; *N* = 23) were generated. The metadata and raw sequences files related to this project were deposited in the NCBI Sequence Read Archive (Accession #SRP059627).

### Data analysis and bioinformatics

Raw sequence reads were first checked using FastQC (http://www.bioinformatics.bbsrc.ac.uk/projects/fastqc/). The effect of trimming of low-quality nucleotides on genome alignment was examined using STAR algorithm^38^. Raw sequence reads (FASTQ files) of 23 samples were mapped against the ovine reference genome Oar_v3.1 using STAR (v2.3.1t) with default parameters. The uniquely mapped read were used to count against the Ensembl annotation Oar_v3.1 for calculating the number of reads per gene. The counts of all samples were tabulated. This table was then inputted to DESeq^39^ for normalization and identification of differentially expressed genes between infection and control groups of both CHS and CS using the standard workflow as described^38^. To correct for multiple hypothesis testing, the Benjamini-Hochberg procedure^40^ was used with an FDR cutoff of 0.05. Gene Ontology (GO) analysis over differentially expressed genes was performed using Fisher’s exact test.

### Real-Time RT-PCR (qPCR) validation

In order to validate the results obtained in the RNAseq analysis, the expression of 7 genes (see Supplementary table for their primer sequences) was determined by qPCR as previously described^36^. Ovine ribosomal protein L19 gene (RPL19), whose expression remained stable among the experimental samples, was used as an endogenous reference gene for all reactions. cDNA was synthesized from high quality total RNA (RIN > 7.5) using Superscript II reverse transcriptase (Invitrogen, Carlsbad, Carlsbad, CA) according to the manufacturer’s instructions. All qPCR reactions were carried out in 96-well plates in a 7500 Real-Time PCR System and analysed with a 7500 Software v2.0.6 (Applied Biosystems, NY). Samples were run in duplicate in a total volume of 25 μl containing the following: 5 μl of cDNA (100 ng), 1 μl of primer mix (forward and reverse, 10 nM each), 0.1 μl of ROX, 12.5 μl of SYBR GreenER qPCR SuperMix (Invitrogen) and 6.4 μl of dd water. The amplification reactions were subjected to a holding stage of 50 °C for 2 min, followed by an initial denaturation at 95 °C for 10 min. The reactions were then followed by 40 cycles of 95 °C for 30 sec, 60 °C for 30 sec and 72 °C for 32 sec. Melting curves were obtained from 60 °C to 95 °C. Relative gene expression values were determined using a standard curve method. Briefly, eight 10-fold serial dilutions of a pool of cDNA samples were used to generate standard curves for each gene to calculate relative gene expression levels. These results were then normalized to RPL19 gene expression levels for each sample.

## Additional Information

**How to cite this article**: Guo, Z. *et al*. Possible mechanisms of host resistance to *Haemonchus contortus* infection in sheep breeds native to the Canary Islands. *Sci. Rep.* **6**, 26200; doi: 10.1038/srep26200 (2016).

## Supplementary Material

Supplementary Information

## Figures and Tables

**Table 1:** Genes significantly impacted by *Haemonchus contortus* infection in both CHB and CS breeds.

**Gene_ID**	**Symbol**	**Fold change**		**FDR**	
		**CHB**	**CS**	**CHS**	**CS**
ENSOARG00000000338	ABCA2	2.86	2.40	2.28%	1.29%
ENSOARG00000008480	ALOX15	10.02	11.45	2.06%	0.00%
ENSOARG00000015249	CDH26	150.19	54.19	0.00%	0.00%
ENSOARG00000018133	CFTR	4.33	2.38	1.21%	3.67%
ENSOARG00000014842	COL6A5	20.79	6.28	1.67%	0.04%
ENSOARG00000007787	FCER1A	20.69	18.23	0.00%	0.00%
ENSOARG00000019163	HBBB	11.47	43.75	1.63%	0.00%
ENSOARG00000008994	IGHE	22.73	35.02	0.00%	0.00%
ENSOARG00000013111	IL1RL1	9.73	5.26	0.00%	0.00%
ENSOARG00000016842	MCTP1	3.89	2.95	1.26%	1.03%
ENSOARG00000002234	SLC2A3	4.39	3.06	0.00%	0.03%
ENSOARG00000005322	SRGN	4.25	3.60	0.45%	0.00%
ENSOARG00000012855	ST3GAL4	2.44	3.69	0.84%	0.94%
ENSOARG00000009990	SYNM	2.16	6.18	2.99%	0.09%
ENSOARG00000005941	TNC	3.00	4.01	2.80%	3.19%
ENSOARG00000014689	TPSAB1	6.96	9.43	0.00%	0.00%
ENSOARG00000006342	TTR	0.28	0.37	0.00%	3.17%
ENSOARG00000000857		8.41	7.84	0.00%	1.29%
ENSOARG00000002036		38.84	44.81	0.00%	0.00%
ENSOARG00000002629		13.34	23.51	0.00%	0.00%
ENSOARG00000002942		7.16	7.83	0.00%	0.00%
ENSOARG00000002964		12.61	29.65	0.01%	0.00%
ENSOARG00000006087		13.49	26.69	0.00%	0.00%
ENSOARG00000013005		3.00	3.53	4.13%	0.94%
ENSOARG00000013263		71.33	91.67	0.00%	0.01%
ENSOARG00000017398		5.73	7.34	2.15%	0.65%
ENSOARG00000023771		0.46	3.00	0.19%	1.55%

**Table 2:** 41 extracellular matrix (ECM) related genes significantly affected by *Haemonchus contortus* infection in the abomasal mucosa of the Canaria Hair Breed sheep (CHB).

**GeneID**	**Symbol**	**Fold change**	***P*** **value**	**FDR**
ENSOARG00000013782	ALB	0.20	0.0000	0.10%
ENSOARG00000008507	ALPL	10.59	0.0000	0.00%
ENSOARG00000005139	APLP1	0.54	0.0001	0.49%
ENSOARG00000018738	BMP2	1.87	0.0004	1.11%
ENSOARG00000012877	CFP	2.78	0.0000	0.19%
ENSOARG00000004871	COL1A1	2.18	0.0005	1.24%
ENSOARG00000001508	COL1A2	1.87	0.0029	4.42%
ENSOARG00000016476	COL3A1	2.09	0.0000	0.21%
ENSOARG00000002129	COL5A1	2.39	0.0002	0.55%
ENSOARG00000016440	COL5A2	1.93	0.0001	0.28%
ENSOARG00000012810	COL6A1	3.72	0.0000	0.00%
ENSOARG00000012880	COL6A2	3.25	0.0000	0.17%
ENSOARG00000019080	COL6A3	2.65	0.0003	0.83%
ENSOARG00000014842	COL6A5	20.79	0.0008	1.67%
ENSOARG00000006410	COL12A1	2.37	0.0005	1.26%
ENSOARG00000009670	CPXM2	2.10	0.0007	1.63%
ENSOARG00000017328	F3	3.03	0.0000	0.06%
ENSOARG00000019404	FBLN1	1.53	0.0022	3.54%
ENSOARG00000017189	FBN2	0.41	0.0000	0.01%
ENSOARG00000016733	FGA	0.38	0.0009	1.93%
ENSOARG00000019329	FN1	4.77	0.0000	0.07%
ENSOARG00000018483	ITGA11	3.37	0.0015	2.75%
ENSOARG00000016642	ITGB7	1.93	0.0032	4.70%
ENSOARG00000010344	LTBP1	2.34	0.0000	0.16%
ENSOARG00000005315	MMP1	11.03	0.0001	0.47%
ENSOARG00000013161	MMP11	0.41	0.0000	0.21%
ENSOARG00000019414	MMP14	1.85	0.0001	0.25%
ENSOARG00000018035	MMP2	1.99	0.0000	0.12%
ENSOARG00000008537	NAV2	0.57	0.0022	3.52%
ENSOARG00000010519	OLFML2B	2.26	0.0004	1.03%
ENSOARG00000006153	PDGFA	0.51	0.0009	1.85%
ENSOARG00000005685	PLOD2	3.06	0.0011	2.15%
ENSOARG00000010041	POSTN	6.73	0.0000	0.00%
ENSOARG00000005275	PXDN	1.94	0.0010	1.98%
ENSOARG00000004813	SDC2	1.95	0.0006	1.39%
ENSOARG00000005209	SDC4	0.57	0.0009	1.93%
ENSOARG00000006391	SERPINB5	3.99	0.0000	0.00%
ENSOARG00000020413	SERPINE2	1.78	0.0003	0.80%
ENSOARG00000015081	TGFBI	3.12	0.0000	0.13%
ENSOARG00000005941	TNC	3.00	0.0016	2.80%
ENSOARG00000008334	VEGFA	0.44	0.0000	0.05%

**Table 3:** 100 cell-cycle related genes significantly affected by *Haemonchus contortus* infection in the resistant breed (CHB).

**Gene ID**	**Locus (chr:start:end)**	**Gene symbol**	**Fold (I/C)**	***P*****value**	**FDR**
ENSOARG00000004361	4:60803768:60856028	ANLN	5.01	5.05E-05	0.0022
ENSOARG00000019052	11:27395740:27400878	AURKB	2.94	7.80E-05	0.0031
ENSOARG00000012875	18:20942195:21007732	BLM	2.46	8.65E-04	0.0185
ENSOARG00000018738	13:48462231:48472599	BMP2	1.87	4.29E-04	0.0111
ENSOARG00000004835	11:42540868:42607539	BRCA1	3.09	6.51E-04	0.0152
ENSOARG00000020126	7:32810618:32861855	BUB1B	3.38	9.57E-05	0.0037
ENSOARG00000020247	7:33203355:33258031	CASC5	3.52	2.94E-03	0.0442
ENSOARG00000014176	6:3657736:3663110	CCNA2	3.95	2.00E-07	0.0000
ENSOARG00000009908	X:51894389:51938870	CCNB3	4.54	1.46E-03	0.0269
ENSOARG00000010352	3:209738381:209761224	CCND2	2.44	1.88E-04	0.0060
ENSOARG00000004318	25:16420008:16437119	CDC2	3.96	3.23E-04	0.0089
ENSOARG00000020542	1:17815061:17818632	CDC20	3.07	5.86E-04	0.0140
ENSOARG00000001274	13:50726485:50734786	CDC25B	1.82	3.24E-03	0.0476
ENSOARG00000014063	11:40114667:40125179	CDC6	4.24	2.32E-08	0.0000
ENSOARG00000009851	2:39869389:39909559	CDCA2	2.74	1.11E-03	0.0218
ENSOARG00000005652	3:207550908:207552937	CDCA3	2.65	2.88E-05	0.0015
ENSOARG00000019830	1:12275289:12289262	CDCA8	3.70	7.07E-05	0.0029
ENSOARG00000011059	6:21798087:21861426	CENPE	3.68	1.30E-05	0.0008
ENSOARG00000012529	12:53214185:53224480	CENPL	2.72	6.91E-04	0.0158
ENSOARG00000008206	14:7100459:7127206	CENPN	3.59	6.95E-05	0.0029
ENSOARG00000003186	14:34675201:34680944	CENPT	2.71	1.54E-03	0.0276
ENSOARG00000007744	8:12047574:12055213	CENPW	3.21	1.43E-04	0.0049
ENSOARG00000003158	22:14531509:14550251	CEP55	4.11	2.07E-06	0.0002
ENSOARG00000009704	5:17254276:17278480	CHAF1A	2.25	1.82E-04	0.0059
ENSOARG00000009289	10:21841538:21861932	CKAP2	3.27	2.54E-03	0.0403
ENSOARG00000007721	2:23987476:23992086	CKS2	3.46	1.08E-06	0.0001
ENSOARG00000019542	1:10450581:10480905	CLSPN	2.00	1.47E-03	0.0269
ENSOARG00000021089	7:64546752:64587073	DLGAP5	3.93	6.21E-04	0.0147
ENSOARG00000017620	1:186409:193575	DTYMK	1.92	2.03E-03	0.0339
ENSOARG00000007334	2:242210461:242229702	E2F2	3.47	4.09E-05	0.0019
ENSOARG00000008807	21:25034831:25052009	E2F8	4.39	3.03E-08	0.0000
ENSOARG00000020622	3:199025742:199151626	EPS8	2.36	6.02E-05	0.0025
ENSOARG00000005908	X:61406551:61410223	ERCC6L	4.54	7.42E-07	0.0001
ENSOARG00000014845	2:100985329:101014711	ESCO2	3.40	5.21E-05	0.0022
ENSOARG00000000644	13:67321805:67342479	FAM83D	3.04	9.58E-05	0.0037
ENSOARG00000005211	19:16642527:16690458	FANCD2	2.84	2.96E-05	0.0015
ENSOARG00000003968	8:76466657:76471151	FBXO5	3.65	7.37E-05	0.0030
ENSOARG00000015633	21:39647404:39648523	FEN1	2.10	9.02E-04	0.0191
ENSOARG00000011054	3:210883196:210893395	FOXM1	3.42	6.81E-04	0.0158
ENSOARG00000007717	13:52921512:52934511	GINS1	3.70	8.22E-06	0.0006
ENSOARG00000004495	22:15498816:15537211	HELLS	3.82	1.95E-09	0.0000
ENSOARG00000019189	1:6959524:6970726	HJURP	3.11	1.52E-04	0.0051
ENSOARG00000020743	7:42727941:42735567	KIAA0101	4.06	1.23E-06	0.0001
ENSOARG00000004780	19:16405186:16463300	KIF15	3.29	2.62E-03	0.0413
ENSOARG00000015211	15:56874125:56943420	KIF18A	2.92	1.68E-03	0.0295
ENSOARG00000015873	5:46994289:47001867	KIF20A	3.56	6.85E-05	0.0028
ENSOARG00000005591	24:26450684:26467845	KIF22	2.77	7.56E-05	0.0030
ENSOARG00000018647	7:16078138:16122235	KIF23	2.92	1.63E-03	0.0288
ENSOARG00000001102	1:19196065:19216520	KIF2C	3.45	2.73E-04	0.0079
ENSOARG00000009637	20:7678567:7686289	KIFC1	2.48	5.81E-04	0.0139
ENSOARG00000020216	7:33016321:33025738	KNSTRN	2.15	1.66E-03	0.0292
ENSOARG00000009349	17:52444861:52510658	KNTC1	2.66	1.04E-03	0.0208
ENSOARG00000015347	11:48337064:48345443	KPNA2	2.30	1.21E-04	0.0044
ENSOARG00000015665	6:5610960:5622911	MAD2L1	2.76	1.99E-04	0.0062
ENSOARG00000005416	13:26912745:26937031	MCM10	2.02	3.35E-03	0.0487
ENSOARG00000014143	20:24477536:24494074	MCM3	2.06	3.46E-05	0.0017
ENSOARG00000012797	9:32400919:32411149	MCM4	2.57	1.32E-06	0.0001
ENSOARG00000018527	3:178690575:178707822	MCM5	2.32	1.72E-05	0.0010
ENSOARG00000010614	2:173834090:173868287	MCM6	2.25	3.91E-05	0.0019
ENSOARG00000011541	2:51686232:51755300	MELK	3.87	1.90E-06	0.0002
ENSOARG00000009575	18:53870504:53915925	MIS18BP1	3.74	3.46E-03	0.0499
ENSOARG00000014562	22:46439178:46468099	MKI67	3.15	6.36E-04	0.0149
ENSOARG00000014901	8:60237288:60271515	MYB	3.06	2.71E-05	0.0014
ENSOARG00000003547	13:71800636:71832436	MYBL2	3.10	1.78E-07	0.0000
ENSOARG00000004016	6:37256547:37333851	NCAPG	3.36	5.28E-06	0.0004
ENSOARG00000007995	4:118730937:118799615	NCAPG2	2.64	6.03E-05	0.0025
ENSOARG00000009604	23:37208584:37244969	NDC80	3.71	4.15E-06	0.0003
ENSOARG00000011466	12:69915815:69927483	NEK2	3.45	4.25E-05	0.0019
ENSOARG00000011189	1:113098396:113135220	NUF2	2.47	9.75E-04	0.0199
ENSOARG00000005282	1:26531082:26561083	ORC1	2.82	4.59E-05	0.0021
ENSOARG00000014858	2:101015056:101043656	PBK	4.32	1.36E-05	0.0008
ENSOARG00000017133	13:46615170:46619285	PCNA	2.05	1.17E-04	0.0043
ENSOARG00000010890	12:52045755:52079590	PDPN	2.08	1.43E-03	0.0264
ENSOARG00000015691	17:29540624:29557094	PLK4	1.97	2.02E-03	0.0338
ENSOARG00000020607	7:39763421:39793166	POLE2	2.83	3.61E-04	0.0096
ENSOARG00000012267	18:20787183:20802103	PRC1	3.88	9.65E-04	0.0198
ENSOARG00000020254	7:33275919:33306843	RAD51	3.12	7.61E-05	0.0031
ENSOARG00000010707	24:15349788:15350439	RAN	1.79	1.72E-03	0.0300
ENSOARG00000017221	13:65583237:65642844	RBL1	2.12	2.02E-03	0.0338
ENSOARG00000017883	13:58047850:58061508	RBM38	2.35	3.13E-03	0.0464
ENSOARG00000015333	3:19185137:19191043	RRM2	5.12	5.48E-09	0.0000
ENSOARG00000017493	6:91484955:91532036	SEPT11	2.18	2.04E-04	0.0063
ENSOARG00000018302	2:239211366:239212113	SFN	2.06	2.68E-03	0.0420
ENSOARG00000007399	2:18870299:18918858	SMC2	2.60	1.89E-03	0.0324
ENSOARG00000001001	11:19659072:19677569	SPAG5	2.45	1.79E-04	0.0058
ENSOARG00000017725	5:13248115:13254108	SPC24	2.98	3.22E-04	0.0089
ENSOARG00000002888	16:1518544:1544794	SPDL1	1.95	2.92E-03	0.0442
ENSOARG00000011578	18:20124947:20166662	TICRR	2.83	1.61E-03	0.0286
ENSOARG00000001419	13:60597535:60651659	TPX2	2.52	6.32E-04	0.0149
ENSOARG00000016302	7:3740229:3789354	TRIM36	10.24	1.19E-14	0.0000
ENSOARG00000007151	8:6885831:6925302	TTK	3.59	9.22E-06	0.0006
ENSOARG00000018884	3:136887716:136929972	TUBA4A	2.02	1.14E-04	0.0042
ENSOARG00000006520	13:74125916:74129104	UBE2C	3.13	1.25E-05	0.0008
ENSOARG00000008530	5:16805815:16842579	UHRF1	3.99	2.60E-07	0.0000
ENSOARG00000004351	8:81449297:81450503		1.84	8.66E-04	0.0185
ENSOARG00000006991	19:59822300:59844027		2.08	2.93E-04	0.0084
ENSOARG00000005764	2:240064407:240068660		2.37	2.89E-05	0.0015
ENSOARG00000006571	21:17310180:17310609		2.66	3.78E-06	0.0003
ENSOARG00000005759	16:10486354:10496192		3.81	1.78E-04	0.0058
ENSOARG00000000647	22:13731293:13771253		3.92	4.05E-04	0.0106

Fold is expressed as infected/uninfected controls. FDR: false discovery rate.

**Table 4:** Gene Ontology (GO) biological processes (BP) significantly enriched in both resistant (CHB) and susceptible (CS) breeds.

**GO_ID**	**Description**	***Z*****Score**		***P*****value**	
		**Resistant**	**Susceptible**	**Resistant**	**Susceptible**
GO:0002250	adaptive immune response	4.65	5.09	4.59E-05	7.37E-04
GO:0046456	eicosanoid biosynthetic process	6.92	6.84	3.81E-07	6.59E-04
GO:0006691	leukotriene metabolic process	7.22	9.67	9.37E-07	9.52E-05
GO:1901570	fatty acid derivative biosynthetic process	6.92	6.84	3.81E-07	6.59E-04
GO:0006636	unsaturated fatty acid biosynthetic process	7.02	6.43	2.12E-07	9.16E-04

**Table 5:** Selected Gene Ontology (GO) terms significantly impacted by *Haemonchus contortus* infection in the resistant breed (CHB).

**GO_id**	**Ontology**	**Description**	**Observed/Total**	**Z Score**	***P*** **Value**
GO:0019369	BP	arachidonic acid metabolic process	10/36	4.95	1.13E-04
GO:0002673	BP	regulation of acute inflammatory response	15/38	7.93	1.16E-08
GO:0002675	BP	positive regulation of acute inflammatory response	8/13	7.78	4.69E-07
GO:0050727	BP	regulation of inflammatory response	24/135	5.01	1.53E-05
GO:0050900	BP	leukocyte migration	30/183	5.11	7.54E-06
GO:0043410	BP	positive regulation of MAPK cascade	30/197	4.65	3.28E-05
GO:0006956	BP	complement activation	14/33	8.06	1.18E-08
GO:0030449	BP	regulation of complement activation	11/23	7.75	9.91E-08
GO:0006957	BP	complement activation, alternative pathway	7/10	7.87	7.26E-07
GO:0006958	BP	complement activation, classical pathway	11/25	7.33	2.87E-07
GO:0031714	MF	C5a anaphylatoxin chemotactic receptor binding	5/5	8.21	1.55E-06
GO:0031715	MF	C5L2 anaphylatoxin chemotactic receptor binding	4/4	7.35	2.25E-05
GO:0007049	BP	cell cycle	119/1137	5.00	1.53E-06
GO:0022402	BP	cell cycle process	97/865	5.21	8.16E-07
GO:0008283	BP	cell proliferation	111/1013	5.34	3.84E-07
GO:0051301	BP	cell division	77/504	7.59	1.83E-11
GO:0051302	BP	regulation of cell division	23/140	4.47	8.26E-05
GO:0051321	BP	meiotic cell cycle	15/87	3.82	8.23E-04
GO:0031577	BP	spindle checkpoint	11/40	5.15	5.72E-05
GO:0007088	BP	regulation of mitosis	15/80	4.20	3.26E-04
GO:0042555	CC	MCM complex	5/11	5.05	5.03E-04
GO:0044818	BP	mitotic G2/M transition checkpoint	6/11	6.24	3.63E-05
GO:0007186	BP	G-protein coupled receptor signaling pathway	33/207	5.18	4.97E-06
GO:0043627	BP	response to estrogen	16/83	4.46	1.48E-04
GO:0001676	BP	long-chain fatty acid metabolic process	11/53	3.99	8.27E-04
GO:0033500	BP	carbohydrate homeostasis	15/81	4.14	3.75E-04
GO:0006865	BP	amino acid transport	14/71	4.27	2.97E-04
GO:0030141	CC	secretory granule	28/172	4.89	1.72E-05
GO:0051048	BP	negative regulation of secretion	12/57	4.22	4.23E-04
GO:0071229	BP	cellular response to acid	19/96	5.00	2.49E-05
GO:0001696	BP	gastric acid secretion	6/7	8.23	7.00E-07

BP = Biological processes. MF = Molecular functions. CC = Cellular components.
